# Advances in 3D printing technology for preparing bone tissue engineering scaffolds from biodegradable materials

**DOI:** 10.3389/fbioe.2024.1483547

**Published:** 2024-11-14

**Authors:** Zhen Wang, Yanan Sun, Chen Li

**Affiliations:** ^1^ College of Mechanical and Electronic Engineering, Shandong University of Science and Technology, Qingdao, China; ^2^ State Key Laboratory of Crane Technology, Yanshan University, Hebei, China; ^3^ Guangdong Provincial Key Laboratory of Minimally Invasive Surgical Instruments and Manufacturing Technology, Guangdong University of Technology, Guangzhou, China; ^4^ School of Information Science and Engineering, Yanshan University, Hebei, China

**Keywords:** bone tissue engineering, biodegradable materials, bone regeneration, 3D bioprinting technology, scaffold fabrication

## Abstract

**Introduction:**

Bone tissue engineering (BTE) provides an effective repair solution by implanting osteoblasts or stem cells into biocompatible and biodegradable scaffolds to promote bone regeneration. In recent years, the rapid development of 3D bioprinting has enabled its extensive application in fabricating BTE scaffolds. Based on three-dimensional computer models and specialized “bio-inks,” this technology offers new pathways for customizing BTE scaffolds. This study reviews the current status and future prospects of scaffold materials for BTE in 3D bioprinting.

**Methods:**

This literature review collected recent studies on BTE and 3D bioprinting, analyzing the advantages and limitations of various scaffold materials for 3D printing, including bioceramics, metals, natural polymers, and synthetic polymers. Key characteristics like biocompatibility, mechanical properties, and degradation rates of these materials were systematically compared.

**Results:**

The study highlights the diverse performances of materials used in BTE scaffolds. Bioceramics exhibit excellent biocompatibility but suffer from brittleness; metals offer high strength but may induce chronic inflammation; natural polymers are biocompatible yet have poor mechanical properties, while synthetic polymers offer strong tunability but may produce acidic by-products during degradation. Additionally, integrating 3D bioprinting with composite materials could enhance scaffold biocompatibility and mechanical properties, presenting viable solutions to current challenges.

**Discussion:**

This review summarizes recent advances in 3D bioprinting for BTE scaffold applications, exploring the strengths and limitations of various materials and proposing composite material combinations to improve scaffold performance. By optimizing material selection and combinations, 3D bioprinting shows promise for creating customized scaffolds, offering a new technical route for clinical applications of BTE. This research provides a unique perspective and theoretical support for advancing 3D bioprinting technology in bone regeneration, outlining future directions for BTE materials and 3D bioprinting technology development.

## 1 Introduction

When patients face significant bone defects caused by severe trauma, infectious diseases, or tumors, surgical bone grafting is often required for complete healing, making bone tissue the second most commonly transplanted tissue today (Migliorini et al., 2021). Traditional autologous or allogeneic bone grafts frequently encounter issues such as donor shortages, immune rejection, and the need for secondary surgeries (Dalipi et al., 2022). Bone tissue engineering (BTE) has the potential to mitigate these problems by promoting rapid bone regeneration. This is achieved by seeding functional cells onto biocompatible scaffolds, which are cultured *in vitro* to maturity before being implanted to facilitate bone regeneration. The implanted scaffold provides a habitat for cells, aiding in nutrient supply, gas exchange, and waste removal. As the material degrades, the implanted bone cells proliferate, ultimately leading to the repair of bone defects (Ellermann et al., 2023; Jia et al., 2021).

The key to BTE lies in identifying scaffold materials that are highly biocompatible, rapidly degradable, non-toxic, and possess excellent porosity and surface bioactivity. Traditional scaffold materials such as bioceramics, glass, metals, and polymers often lack bioactivity, leading to issues like poor integration, wear, and corrosion, thus hindering functional bone regeneration (Deng et al., 2023; Abbas et al., 2021; Pazarçeviren et al., 2021). While composite materials have addressed some of the limitations of single materials, challenges like manufacturing complexity, brittleness, and susceptibility to aging continue to impede the development of BTE (Cannillo et al., 2021).

3D printing technology constructs objects by layering adhesive materials, such as powdered metals or plastics, based on digital model files (Yang, 2022). This technology simplifies and accelerates the fabrication of bone tissue engineering scaffolds, significantly reducing production time while enabling the creation of personalized scaffolds with complex structures, which greatly benefit patient injury repair (Anandhapadman et al., 2022). The rapid development of 3D bioprinting, in particular, has positioned it as one of the most promising technologies for producing tissue engineering scaffold materials, with the potential to address major challenges in material preparation and drive rapid advancements in materials science and medicine (Liu et al., 2022). In recent years, the application of low-temperature printing technology has further enhanced scaffold performance. Gao et al. (2022) demonstrated that hierarchically porous scaffolds produced through low-temperature printing exhibit significant advantages in biomineralization and bone regeneration. Although existing review articles extensively discuss the applications of 3D bioprinting in bone tissue engineering, most focus primarily on material selection and process optimization, with limited in-depth analysis of the challenges and potential barriers to clinical application. These reviews often overlook how 3D bioprinting, when combined with innovative biomaterials and personalized structural designs, can address current challenges in bone tissue engineering. In response, this paper provides a comprehensive summary of the clinical applications of 3D bioprinting, analyzing issues such as the controllable degradability of printing materials, mechanical compatibility with bone tissue, and post-implantation biocompatibility. Additionally, the paper explores how innovative material compounding techniques and structural optimization can improve the clinical applicability of 3D-printed scaffolds, addressing gaps in the existing research on practical applications.

## 2 Types of bone tissue engineering scaffold materials

**FIGURE 1:**
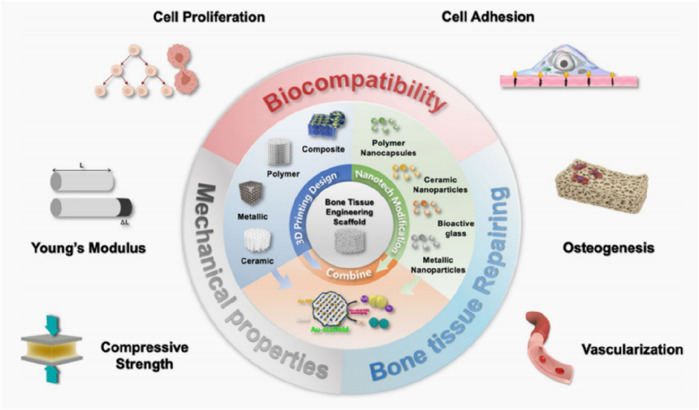
3D printing technology of bone tissue engineering templates. (Wu et al., 2024).

As one of the most important load-bearing tissues in the human body, the selection of materials for bone tissue engineering must balance load-bearing capacity, biocompatibility, and degradability. Firstly, the materials should match the mechanical properties of bone, providing sufficient strength and toughness to offer support and avoid stress shielding (Mahmoud et al., 2021). Secondly, the materials must have excellent biocompatibility, avoiding immune rejection while possessing appropriate porosity and surface activity to promote cell adhesion and vascularization (Sari et al., 2021). Most importantly, the materials should be biodegradable, with a degradation rate that matches the pace of tissue regeneration, and the degradation products must be non-toxic (Llamas-Unzueta et al., 2021). The 3D printed bone tissue engineering strategy is shown in Figure 1, the strategy for bone injury repair using bone tissue engineering involves a wide range of materials. These materials can generally be classified into four categories: inorganic non-metallic materials, metallic materials, organic polymer materials, and composite materials.

### 2.1 Inorganic non-metallic materials

**FIGURE 2:**
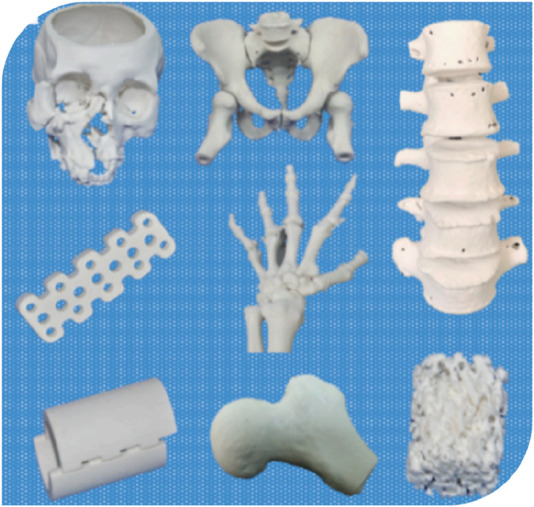
Bioceramic printed bone models and scaffolds. (Wei et al., 2023).

Bioactive ceramics are widely used inorganic non-metallic materials due to their excellent biocompatibility, often referred to as biodegradable ceramics. As shown in Figure 2, Wei et al. (2023) demonstrated bone models and scaffolds printed by bioactive ceramics. Common bioactive ceramic materials include hydroxyapatite (HA), tricalcium phosphate (TCP), biphasic calcium phosphate (BCP), and silicate bioactive ceramics (Alnujaym et al., 2022).

HA is a bioactive material composed of calcium and phosphorus, resembling the inorganic components of human bone. Its porous structure facilitates the adhesion, proliferation, and differentiation of bone cells, and it forms strong bonds with bone tissue after implantation, making it an ideal material for repairing bone defects (Huang et al., 2021). HA addresses the limitations of autografts and the risk of allograft rejection, and it is widely used in hard tissue repair (Tsai et al., 2021). TCP is divided into the high-temperature phase α-TCP and the low-temperature phase β-TCP. β-TCP is non-cytotoxic, capable of withstanding normal loads after implantation, and offers good biocompatibility, osteoconductivity, and degradability, making it commonly used in bone regeneration (Lu et al., 2021). In physiological environments, β-TCP degrades rapidly, releasing calcium (Ca) and phosphorus (P) into the body’s circulatory system (Lee et al., 2021). BCP, composed of hydroxyapatite (HA) and tricalcium phosphate (TCP), allows the degradation rate and osteoconductivity to be controlled by adjusting the ratio of HA to TCP. BCP combines the stability of HA with the degradability of TCP, offering excellent biocompatibility and osteoconductivity. Compared to single materials, BCP is better suited to meet diverse bone regeneration needs under different physiological conditions, providing both long-term bone support and releasing calcium and phosphorus through TCP degradation to promote new bone formation. Moreover, the degradation rate of BCP can be tailored to specific repair needs, optimizing its bioactivity, mechanical strength, and controlled degradation properties. Research on silicate bioactive ceramics started relatively recently, but they have gained attention for their ability to release silicon ions. Silicon, an essential trace element, is closely related to bone quality, especially during early bone development, as it promotes early bone calcification (Riccardi et al., 2021). Therefore, as scaffold materials for bone tissue engineering, silicate ceramics significantly enhance bone cell proliferation, differentiation, and repair (Li et al., 2021).

### 2.2 Metallic materials

Metallic materials, due to their high mechanical strength and favorable elastic and plastic properties, have long been used as orthopedic implants. Currently, widely used metal materials include stainless steel, titanium alloys, and cobalt alloys. These materials serve as permanent implants; however, long-term implantation in the human body can lead to complications such as stress shielding, metal ion release, and chronic inflammation (Tsakiris et al., 2021; Zhang E. et al., 2021; He et al., 2023). Among these, magnesium and titanium are widely applied in bone tissue engineering scaffolds.

**FIGURE 3:**
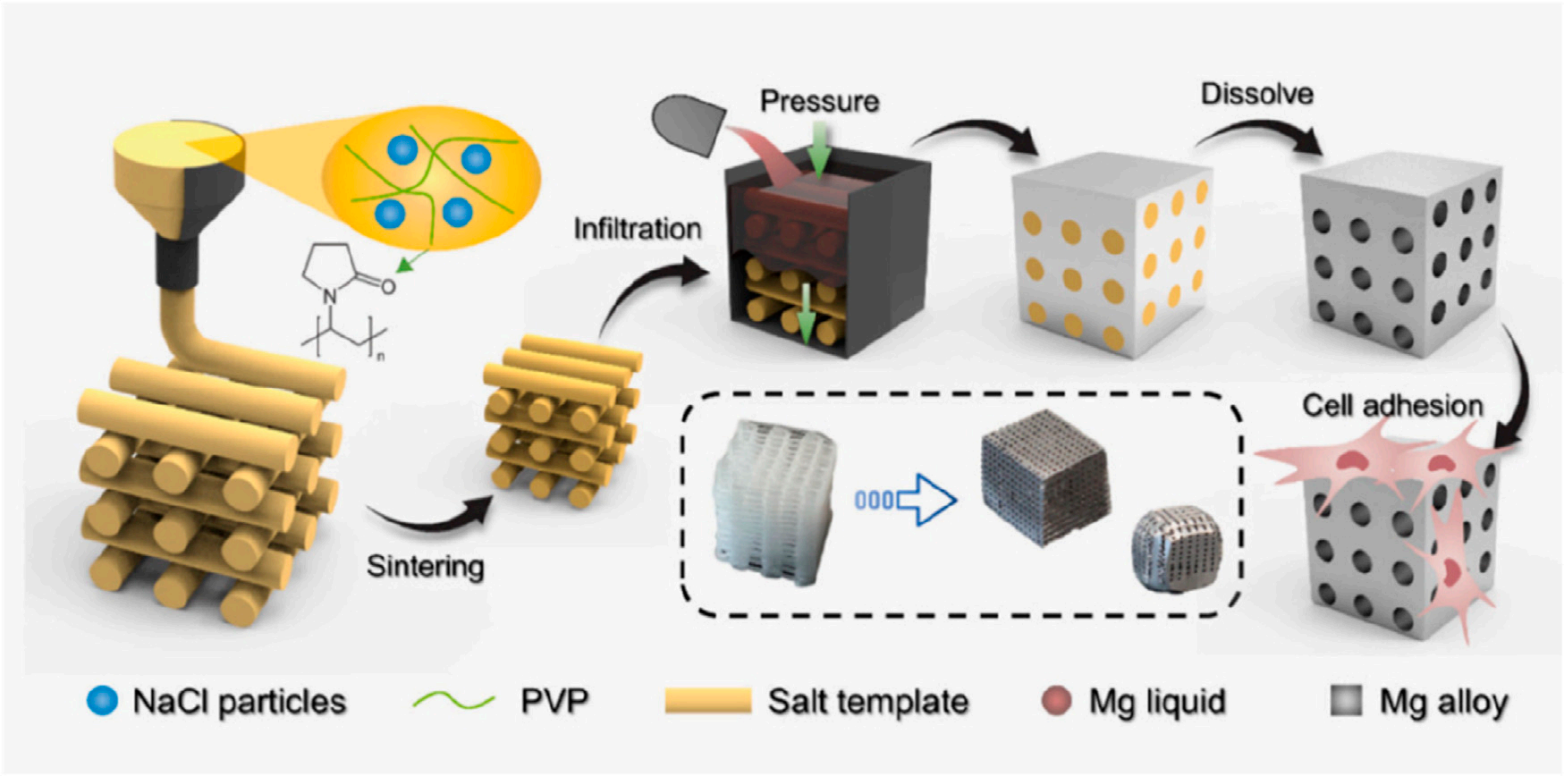
Process of 3D printing salt template and magnesium infiltration preparation. (Li et al., 2024).

Magnesium is an essential nutrient for the human body, playing a critical role in activating various enzymes, stabilizing DNA and RNA structures, and supporting nerve, muscle, bone, and heart function. Approximately half of the body’s magnesium is stored in bone tissue (Zhou et al., 2021). As shown in Figure 3, scholar Li et al. (2024) demonstrated the process of preparing bone tissue engineering scaffold by 3D printing salt template and impregnating metal magnesium. Magnesium’s density and elastic modulus are similar to those of human bone, and it gradually degrades into magnesium ions that are either absorbed or excreted from the body, making it an excellent biodegradable material (Jhamb et al., 2021). Studies have shown that pure magnesium and magnesium alloys are non-cytotoxic, non-genotoxic, and free from acute systemic toxicity, with good biocompatibility (Zhi et al., 2022). Titanium, known for its superior mechanical properties, elastic modulus, and corrosion resistance, also exhibits high biocompatibility and has gradually found clinical application. As an orthopedic replacement material, titanium and its composites improve integration with surrounding bone tissue, enhance osteoblast function, and promote bone regeneration (Wu et al., 2021). Traditional scaffold materials often suffer from poor compatibility and inability to adapt to individual growth, which can compromise the repair outcomes (Yazdanpanah et al., 2022). Titanium, on the other hand, is easy to manipulate, elicits minimal rejection, and is highly malleable, allowing it to bond tightly with the host bone post-implantation, resulting in significant bone defect repair outcomes (Sarraf et al., 2021).

### 2.3 Polymer materials

Organic polymer materials can be categorized into two types: natural and synthetic. Natural polymers are derived from animals, plants, human tissues, or synthesized by microorganisms. They exhibit good biocompatibility and provide cell recognition signals, aiding in cell adhesion, proliferation, and differentiation (Jurak et al., 2021). Examples include protein-based materials such as gelatin, silk fibroin, and collagen, as well as polysaccharides like chitosan and alginate. However, natural polymers have several limitations, including susceptibility to microbial contamination, potential immune reactions, uncontrollable degradation rates, and poor mechanical strength, which restrict their application in hard tissue regeneration (Reddy et al., 2021).

Artificially synthesized polymers can be designed with specific compositions, structures, mechanical properties, and degradation rates to meet diverse requirements, making them widely used in tissue engineering scaffolds. Common synthetic biodegradable polymers primarily include bio-polyesters such as polylactic acid (PLA), polyglycolic acid (PGA), poly (lactic-co-glycolic acid) (PLGA), polycaprolactone (PCL), and polyhydroxybutyrate (PHB). Due to their excellent biodegradability and biocompatibility, these materials have gained significant attention in bone regeneration and have been approved by agencies such as the U.S. Food and Drug Administration (FDA) for use in biomedical materials (Gillman and Jayasuriya, 2021). These materials gradually degrade in the body, leaving no residues or toxic byproducts. Their degradation rates and mechanical properties can be tailored by adjusting molecular weight, polymerization methods, and molding techniques, allowing for standardized mass production (Rosli et al., 2021). However, polyesters lack osteoinductive properties, and the release of hydrogen ions during degradation may lower the pH at the implantation site, leading to inflammatory responses. To address this, they are often combined with bioceramics (Zhang H. et al., 2023).

With technological advancements, researchers have developed novel copolymers, such as poly (lactic acid)-poly (caprolactone) (PLA-PCL), poly (lactic-co-glycolic acid)-polyethylene glycol (PLGA-PEG), and trimethylene carbonate-co-lactic acid (PTMC-LA). These materials retain the advantages of traditional polyesters while optimizing performance through copolymerization. Novel copolymers not only provide stable mechanical support but also offer tunable degradation rates, meeting the needs of bone tissue regeneration. As they degrade in the body, they release bioactive molecules that promote bone cell adhesion, proliferation, and differentiation, thus facilitating new bone formation. Compared to traditional materials, these new copolymers offer superior mechanical properties and controlled degradation timing, synchronizing with the bone healing process. In bone regeneration, these new copolymers are widely used to fabricate porous bone tissue scaffolds, providing space for bone cell proliferation and migration through 3D printing and other technologies. Their plasticity and controlled degradability offer long-term support, avoiding the risk of secondary surgeries associated with metal implants. Additionally, these novel copolymers are non-toxic and do not induce inflammation during degradation, ensuring excellent biocompatibility, making them ideal materials for bone tissue engineering (Kirillova et al., 2021), the organic polymer materials used in bone tissue engineering and their advantages and disadvantages are shown in Table 1.

**TABLE 1:** Advantages and disadvantages of common organic polymer materials.

Polymer material		Advantages	Disadvantages	References
Natural Biomaterials	Collagen	Enhances cell function and adhesion, interacts with elastin fibers, provides recoil for extracellular matrix and fibronectin	Poor mechanical properties, potential issues like thrombosis, contamination, and variability in sources and batches, fast degradation rate	Bahrami et al., 2021; Meyer et al., 2023
	Gelatin	Excellent biocompatibility and non-immunogenicity	Poor mechanical properties, fast degradation rate	Dou et al. (2021)
	Silk Fibroin	Excellent biocompatibility, controllable degradation rate, superior mechanical properties	Lacks cell adhesion sites, degradation products may cause immune or inflammatory reactions, complex processing, high cost	Li and Sun (2022)
	Alginate	Non-toxic, good biocompatibility, biodegradability, and hydrophilicity; can be used under mild conditions	Poor mechanical properties, insufficient bioactivity of degradation products, unstable biocompatibility	Fu et al. (2022)
	Hyaluronic Acid	Good biocompatibility, inherent dual functionality, non-immunogenicity, multifunctionality, and biodegradability	Poor mechanical properties, insufficient bioactivity of degradation products, high cost	Sahoo and Biswal (2021)
	Chitosan	Good biocompatibility, biodegradability, antibacterial properties, and mechanical performance	Fast degradation rate, relatively complex preparation process	Ma et al. (2022)
Synthetic Biomaterials	PLA	Biocompatibility, processability, and printability	Releases acidic byproducts, brittle	Shchenko et al., 2023
	PGA	Chemical adaptability, biocompatibility, and biological properties, easy to handle	Rapid erosion can lead to scaffold collapse, releasing acidic degradation products that may affect the body	
	PCL	Lower cost, rigidity, biocompatibility, and biodegradability	Long biological half-life can cause issues within scaffolds, high hydrophobicity leads to low bioactivity	
	PLA-PCL	Combines the benefits of PLA and PCL, offering good biocompatibility and mechanical properties	Complex production process, high cost, may release acidic byproducts during degradation	Wang et al. (2022)
	PLGA-PEG	Excellent biocompatibility, tunable degradation rate, enhanced solubility, suitable for long-term drug delivery and bone scaffolds	The PEG component can sometimes cause excessive hydration, leading to faster-than-expected degradation	Dai et al. (2021)
	PTMC-LA	Non-toxic degradation products, good mechanical strength, suitable for long-term implants, excellent biocompatibility	Slow degradation rate, may require additional treatments to accelerate degradation in some cases	He et al. (2021)

### 2.4 Composite material

**FIGURE 4:**
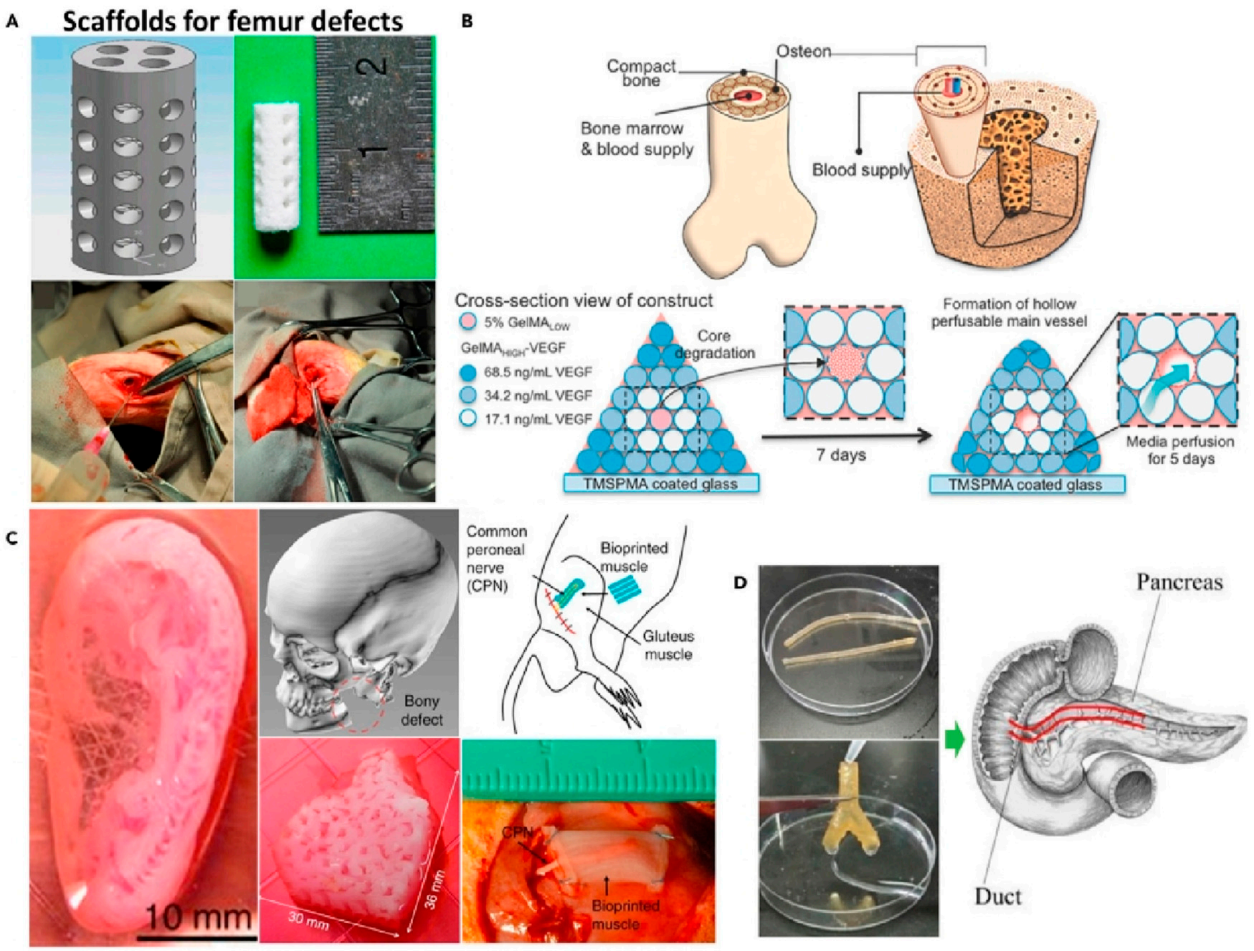
3D printing applications of composite materials. **(A)**. Upper: CAD image of a defective femur stent. Lower: image of the rabbit femur defect. **(B)**. Upper: Complex bone structure diagram. Lower: Schematic structure of bioprinting to create complex bone tissue. **(C)**. Images of bioprinted products implanted in human and rat ear, mandible and muscle defects (from left to right). **(D)**. Chitosan catheter for soft tissue produced by FDM. (Park et al., 2022).

Single materials, due to their inherent limitations, struggle to meet the diverse requirements of bone tissue engineering. While inorganic materials possess excellent biocompatibility and osteoinductivity, they tend to be brittle and fragile, and degrade slowly in the body (Ielo et al., 2022). On the other hand, organic polymers, though biodegradable and absorbable, have poor mechanical properties, their structure differs significantly from human bone tissue, and their degradation byproducts can accumulate, forming an acidic environment that hinders tissue healing (Ilhan et al., 2022). By combining multiple materials, it is possible to leverage their strengths, enhancing mechanical strength, optimizing degradation rates, and improving bioactivity. Therefore, the selection and preparation of composite materials have become a key focus in bone tissue engineering research, 3D printing applications of composite materials are shown in Figure 4. Among them, Figure 4A shows a 3D printed structure for bone tissue construction that contains osteogenic and angiogenic components that promote the formation of a stable blood vessel network. Figure 4B shows an image of a porous cylindrical bone tissue engineering scaffold. Figure 4C shows printed examples of biological tissues such as ears, mandibles, and muscles. Figure 4D shows a chitosan-based catheter manufactured by extrusion 3D printing technology.

The degradation time of composite materials can be flexibly adjusted based on patient needs. By varying the ratio of organic polymers to inorganic materials, the scaffold’s degradation rate can be aligned with the rate of bone regeneration. For example, using a fast-degrading polymer like polylactic acid (PLA), the scaffold can degrade within 6–12 months. By adding slower-degrading materials like tricalcium phosphate (TCP), the scaffold’s functional lifespan can be extended to 1–2 years. For patients with faster bone regeneration, materials with faster degradation rates can be selected, while for patients with slower bone regeneration or those requiring long-term support, more durable materials can be used to extend scaffold longevity. This personalized adjustment not only improves mechanical performance but also allows for more precise treatment. Different bone regeneration environments require varying mechanical properties from scaffolds. In high load-bearing areas (such as leg bones, hips, and the spine), scaffolds require higher mechanical strength and compressive modulus, typically achieved by combining metals with ceramics to ensure structural stability under prolonged high loads. In contrast, in low load-bearing areas (such as the skull and facial bones), lighter polymers or highly bioactive composites are used to provide necessary support while accelerating degradation to promote bone regeneration. As bone tissue forms, the mechanical demands on the scaffold decrease, so material selection and design should be dynamically adjusted to accommodate the different stages of bone regeneration. Based on material selection, composite scaffolds can be categorized into homogenous and heterogeneous types. Homogenous scaffolds include natural polymers, synthetic polymers, and inorganic materials. Heterogeneous scaffolds combine natural polymers with inorganic materials, synthetic polymers with inorganic materials, or metallic-based composites. Among these, porous composites combining inorganic non-metallic and organic polymers are highly promising in bone tissue engineering due to their superior bioactivity and mechanical properties (Li et al., 2022). Organic polymers enhance the toughness of inorganic materials, satisfying mechanical demands, while inorganic components induce osteogenesis and buffer the acidic environment caused by polymer degradation (Ali et al., 2022). The extracellular matrix (ECM) plays a crucial role in tissue repair, providing physical support, promoting cell adhesion, regulating signal transduction, and encouraging angiogenesis. As a result, researchers are increasingly focused on mimicking the structure and function of ECM. Materials should possess a three-dimensional porous structure similar to that of ECM, promoting cell migration and vascularization, and enhancing integration with surrounding tissues by adjusting pore size and porosity. Surface functionalization can enhance cell adhesion, while mimicking the biochemical properties of ECM to stimulate osteoblast differentiation and endothelial cell migration. Natural materials like collagen and chitosan have shown potential in replicating ECM functions. Additionally, embedding bioactive factors that promote angiogenesis or designing intelligent scaffold systems to gradually release growth factors can further enhance vascularization. These strategies not only accelerate bone tissue integration but also improve the speed and quality of tissue repair (Karamanos et al., 2021).

However, it is often difficult to combine all the desired properties in a single material composite, and to further enhance the mechanical strength and functionality of the materials, carbon nanotubes (CNTs) have been introduced as a modified reinforcing agent in recent years.CNTs are widely used in composites due to their unique nano-structures and excellent mechanical and electrical properties, especially in the preparation of bone tissue-engineering scaffolds, where they have demonstrated significant enhancement effects.The modification of carbon nanotubes (CNT) has become a key direction in enhancing bio-ink performance. Due to their nanoscale structure and unique physicochemical properties, CNTs introduce nanoscale changes to 3D-printed scaffolds. Firstly, the high mechanical strength and toughness of CNTs significantly improve the compressive and tensile strength of scaffolds, enhancing their stability. Secondly, CNTs’ nanostructure provides fine surface roughness, promoting cell adhesion and proliferation. Additionally, CNTs’ conductivity helps form conductive networks, facilitating electrical signal transmission, making them suitable for nerve and muscle tissue regeneration. Their nanoscale properties also optimize the rheological performance of bio-inks, ensuring precise control over scaffold microstructures and improving overall functionality (Amiryaghoubi et al., 2021).

When selecting and designing bone regeneration scaffold materials, patient-specific factors such as age and comorbidities play a critical role. Elderly patients, for instance, experience slower bone regeneration and thus require scaffolds with longer degradation times and higher mechanical strength to provide sustained support. Common issues like osteoporosis necessitate materials with strong compressive strength and osteogenic properties, such as calcium phosphate ceramics or calcium-containing composites. For patients with metabolic conditions like diabetes, who often have impaired healing abilities, scaffolds must promote vascularization. Bioactive materials containing growth factors or scaffolds with porous designs can accelerate regeneration. Additionally, scaffold degradation products must be non-toxic and easily metabolized to avoid adverse reactions. Therefore, bone regeneration scaffold materials and designs should be personalized according to the patient’s specific conditions to achieve the best outcomes (Chen et al., 2021).

In scaffold design, a precise balance must be achieved between the degradation rate of the material and its mechanical support capacity, as bone tissue regeneration is a gradual process. In the early stages, the scaffold must provide sufficient mechanical strength to prevent collapse or deformation in the defect area. As new bone forms, the scaffold should gradually degrade to avoid excessive residual material that could interfere with natural bone regeneration. To achieve this dynamic balance, the scaffold should offer adequate initial mechanical support to bear external loads while gradually degrading as bone tissue proliferates, simultaneously releasing bioactive factors that promote bone regeneration. Through material composites, scaffolds can be designed with multilayered structures or gradient degradation properties. The outer layer of the scaffold can be engineered to degrade quickly, promoting early cell proliferation and vascularization, while the inner layer provides long-term mechanical support, ensuring the scaffold does not degrade prematurely before full bone restoration. By synchronizing the scaffold’s degradation rate with its mechanical performance, a balance between rapid degradation and long-term support can be achieved, optimizing bone regeneration outcomes (Wang et al., 2022).

Porosity is a critical factor in scaffold design, influencing both mechanical strength and cell proliferation. A higher porosity increases internal space, facilitating cell migration, proliferation, and the diffusion of nutrients, thereby accelerating bone tissue repair. However, excessively high porosity can compromise the scaffold’s mechanical strength, making it unable to provide adequate support. Therefore, scaffold design must optimize porosity to strike a balance between mechanical strength and cell proliferation. By precisely controlling the pore size, shape, and distribution, it is possible to enhance cell proliferation while maintaining appropriate mechanical performance. Larger pores support cell infiltration and vascularization, while smaller pores improve mechanical strength. Typically, an optimal porosity between 60% and 80% provides sufficient space for cells while ensuring that mechanical performance does not decline significantly. With the help of computer-aided design (CAD), the pore structure of scaffolds can be precisely controlled to meet specific mechanical and biological performance requirements (Mohammadi et al., 2021).

Any foreign material implanted into the body carries the potential to trigger an immune response, particularly through its degradation products, surface characteristics, and interactions with surrounding tissues, which may lead to inflammation or even immune rejection. Inorganic materials, such as calcium phosphates or degradation products of certain metals, may provoke acute or chronic inflammation, while the acidic degradation products of polymers could stimulate the immune system. To minimize immune reactions, scaffold material design must prioritize biocompatibility. First, low-immunogenicity materials, such as calcium phosphate-based bioceramics or biodegradable polymers (like polylactic acid and polyhydroxy acids), should be preferred. In addition, surface modifications, such as bioactive coatings or functionalized surfaces, can enhance integration with surrounding tissues and reduce the activation of immune cells. The pore structure of scaffolds also affects the immune response, with larger, evenly distributed pores promoting tissue infiltration and vascularization, thereby lowering the risk of inflammation. Moreover, the degradation rate of the material should match the pace of tissue regeneration, avoiding rapid degradation that may cause inflammation or slow degradation that might lead to a foreign body reaction. By optimizing these design parameters, scaffold materials can effectively reduce the occurrence of immune responses and improve therapeutic outcomes.

## 3 Preparation method of bone tissue engineering scaffold

Traditional bone tissue engineering scaffold fabrication methods, such as electrospinning, solvent casting-particulate leaching, phase separation, and gas foaming, are commonly used but are limited to producing relatively simple scaffold structures. These scaffolds often suffer from issues such as irregular structures, poorly controlled pore sizes, low mechanical strength, and poor reproducibility, which significantly impact their practical application (Abdelaziz et al., 2023; Chinnasami et al., 2023). Since its first report in 1989, 3D printing technology has been widely applied across various fields, and it has seen rapid development in the fabrication of bone tissue engineering scaffolds, significantly overcoming the limitations of traditional methods. As 3D printing technology has advanced, 3D bioprinting using biomaterials and their derivatives as bio-inks has emerged. Due to their inherent biological origin, these materials offer superior cell proliferation and biocompatibility compared to synthetic polymers (Lin et al., 2022).

3D bioprinting utilizes CT and MRI technologies to obtain tissue samples and medical images of organs, which are then reconstructed using CAD software to generate G-code files for printing. CT is especially suited for accurately capturing bone structure details due to its high resolution and rapid imaging capabilities, clearly displaying the geometric shape of bone defects, making it a crucial tool for designing 3D-printed scaffolds (Chakraborty et al., 2022). Meanwhile, MRI provides excellent soft tissue imaging, supplementing the precise data of the soft tissues surrounding the bone, allowing for better consideration of soft tissue integration and compatibility in scaffold design. By combining these imaging techniques, the three-dimensional shape of bone defects can be mapped comprehensively and accurately, ensuring that 3D-printed scaffolds fit the patient’s specific needs in both size and shape. These imaging techniques allow 3D bioprinting to create custom-made scaffolds tailored to the patient’s individual bone morphology, defect location, and repair requirements. 3D printing technology enables precise control over scaffold shape, structure, and material composition, optimizing degradation rates and mechanical properties (Hanxu et al., 2021). For patients with faster bone regeneration, materials with faster degradation rates can be used, while for those with slower bone regeneration or requiring long-term support, scaffold durability can be enhanced by using more durable materials. Such personalized scaffolds not only match the patient’s bone shape but also allow the degradation rate and mechanical strength to be adjusted through material selection and combination, leading to better therapeutic outcomes. Finally, the 3D bioprinting process includes the isolation, proliferation, and cultivation of cells. Cells are mixed with special liquid materials, which are then transferred to the corresponding printing system based on the material and printing method. Following a preset program, the scaffold is printed layer by layer, ensuring precise integration of cells and materials, promoting bone tissue regeneration and repair.

The application and development of 3D bioprinting technology have brought the fabrication of bone tissue engineering scaffolds to new heights. Currently, common 3D bioprinting technologies include inkjet bioprinting, extrusion-based bioprinting, and laser-assisted bioprinting (Vieira et al., 2021). The primary biomaterials used for 3D printing are polymers, bioceramics, and composite materials (Zhang Q. et al., 2023). However, the scalability of bioprinting methods is a crucial consideration for their successful clinical application. Although 3D bioprinting has demonstrated promising performance at the laboratory scale, producing scaffolds with intricate structures, it faces numerous challenges when scaling up for mass production (Lindner and Blaeser, 2022). First, the speed and scale limitations of current printing equipment make it difficult to produce large quantities of scaffolds, especially for complex and larger structures where the printing time increases significantly, leading to reduced production efficiency. Second, standardizing bio-ink formulations and ensuring batch-to-batch consistency remain significant hurdles. Maintaining consistent cell viability and material performance across different batches of bio-ink is one of the major challenges in achieving large-scale production. Furthermore, maintaining printing precision and fidelity in mass production can be difficult, particularly when designing the scaffold’s internal microstructure. Achieving higher production efficiency without sacrificing precision is a key issue that needs to be addressed. In the future, 3D bioprinting technology is expected to overcome these obstacles by introducing automated systems, multi-nozzle designs, and intelligent material management systems. By enhancing printing efficiency, optimizing bio-ink formulations, and improving process stability, bioprinting technology will be able to achieve high-precision mass production, paving the way for widespread clinical application.

## 4 Application of 3D bioprinting technology in bone tissue engineering

Bone tissue engineering aims to guide cell differentiation and form new functional tissue to replace damaged areas. One of its primary challenges is the development of three-dimensional biodegradable porous structures capable of withstanding mechanical loads and providing mechanical stimulation. With advancements in 3D scanning, design software, and printing technologies, 3D bioprinting has become increasingly integrated into bone tissue engineering. By utilizing live cells and biomaterials as “bio-inks,” bioprinted bionic scaffolds hold great potential for replacing human structures.

In different bone regeneration regions, the design of 3D bioprinted scaffolds must account for variations in the biomechanical environment. In high-load-bearing areas such as the lower limbs and spine, scaffolds must not only have sufficient mechanical strength but also withstand repeated dynamic loading. To avoid damage caused by localized stress concentrations, scaffolds often employ complex porous structures to evenly distribute stress. In these areas, compressive strength is the key design consideration. On the other hand, in low-load-bearing regions like the skull and facial bones, the focus is on the flexibility and morphological adaptability of the scaffold to ensure proper integration with surrounding soft tissue and promote rapid bone regeneration. Additionally, 3D bioprinting offers the flexibility to adjust scaffold properties at different stages of bone regeneration. In the early stages, higher mechanical support is required, and as new bone forms, the mechanical demands on the scaffold decrease. Scaffold designs can incorporate gradual degradation, releasing growth factors and other bioactive substances to accelerate the regeneration process (Zhang X. et al., 2021).

Scaffolds prepared using 3D bioprinting technology, with the addition of osteogenic cells and other biomaterials, significantly enhance osteogenic activity and promote bone regeneration. Maturavongsadit et al. used bio-inks composed of chitosan and cellulose nanocrystals (CNC) and found that the addition of CNC and MC3T3-E1 cells significantly improved the scaffold’s viscosity and mechanical properties, promoting osteogenic differentiation (Maturavongsadit et al., 2021). Han et al. developed a magnetic scaffold by coating poly (lactic-co-glycolic acid) (PLGA) scaffolds with iron oxide nanoparticles (IONPs), which enhanced cell adhesion and new bone formation (Han et al., 2021). Sun et al. successfully printed an artificial periosteum using composite bio-inks containing bone marrow mesenchymal stem cells, demonstrating good thermosensitivity and osteogenic activity (Sun et al., 2023).

In addition to loading osteogenic cells or bioactive factors, bio-inks can also carry antibiotic drugs, creating antimicrobial repair scaffolds that reduce infection risk and the complications associated with bone defect treatments. Mulazzi et al. demonstrated that MgHA/Collagen scaffolds combined with antibiotics could serve as a safe and effective local drug delivery system, preventing infections related to orthopedic surgeries (Mulazzi et al., 2021). Similarly, Zhang et al. developed a 3D-printed scaffold combined with hydrogels that exhibited both antimicrobial and osteogenic properties, showing potential applications in treating infectious bone defects (Zhang S. et al., 2023). The future development of bio-inks will focus on smart drug delivery, multifunctional material integration, and personalized medicine. Smart drug delivery systems could autonomously adjust the release rate and dosage of drugs in response to changes in the body’s microenvironment, such as pH, temperature, or concentrations of inflammatory markers, thereby improving treatment precision and effectiveness. The development of multifunctional materials will push for the integration of properties like antimicrobial activity, tissue repair promotion, and enhanced mechanical strength into a single scaffold. Combining materials that support antibacterial action, osteogenesis, and angiogenesis into one scaffold can better address the multifaceted needs of bone regeneration. Furthermore, personalized medicine will steer the development of composite materials. By combining 3D printing technology with patient-specific data, custom-designed scaffolds can be created to precisely fit the shape and size of the patient’s bone defects, maximizing treatment efficacy and reducing complications.

3D bioprinting provides personalized solutions for various types of bone injuries, particularly excelling in the repair of critical-sized defects. These defects often surpass the body’s natural healing capacity, and traditional methods struggle to address them effectively (Hao et al., 2022). With precision design, 3D-printed scaffolds can not only fully fill defect areas but also provide mechanical support to promote bone cell attachment and proliferation. Common materials like hydroxyapatite and calcium phosphate, which are biocompatible and enhance bone regeneration, are widely used in these scaffolds. In fracture repair, 3D-printed scaffolds can be customized according to the fracture morphology, ensuring a perfect fit with the fracture site and reducing postoperative complications. Moreover, integrating growth factors or drug-delivery systems into the scaffolds can accelerate healing and minimize the need for multiple surgeries. For subchondral bone regeneration, 3D-printed gradient porous scaffolds meet the mechanical support and bioactivity requirements, promoting the simultaneous regeneration of cartilage and bone tissues. In cranial and facial bone reconstruction, 3D bioprinting demonstrates advantages in personalized and complex structure formation, accurately replicating the patient’s bone morphology to ensure synchronized recovery of both function and appearance, while reducing the risks of rejection and secondary surgeries. Ma et al. (2021) showed successful applications of 3D printing in customized complex craniofacial bone reconstruction, further confirming the broad potential of this technology. Overall, 3D bioprinting plays a pivotal role in various bone injury repairs and holds the promise of providing more solutions for complex bone tissue regeneration in the future.

Beyond bone injuries, treating bone tumors and postoperative repair remain clinical challenges. Research shows that bioactive scaffolds prepared via 3D bioprinting have achieved significant success in addressing malignant bone tumors. Li et al. developed magnesium oxide/poly (L-lactide) nano-composite scaffolds using low-temperature 3D printing technology, which release magnesium ions and reactive oxygen species in a controlled manner. This not only prevents tumor recurrence but also inhibits bacterial infection and promotes bone defect repair, offering a novel strategy for post-sarcoma surgery treatment (Li et al., 2023). Yao et al. created composite scaffolds using hydroxyapatite, polydopamine, and carboxymethyl chitosan that promote osteogenic differentiation of bone marrow stromal cells while inducing tumor cell apoptosis and necrosis through photothermal effects, showing great potential in both osteogenesis and bone tumor treatment (Yao et al., 2021).

3D bioprinting technology still faces limitations in terms of resolution and precision, particularly when manufacturing complex, delicate scaffold structures. The resolution of current equipment struggles to achieve the micron-level precision required to replicate the ECM (Ning et al., 2021). This limitation affects the precise control of internal scaffold porosity, which in turn influences the distribution and attachment of cells within the scaffold. Low-resolution printing methods may result in uneven cell distribution and incorrect pore sizes, hindering the diffusion of nutrients and oxygen, and ultimately reducing the efficiency of bone regeneration. With advancements in micro- and nanofabrication technologies, printing resolution is gradually improving. Advanced optical projection printing techniques, such as digital light processing (DLP), are now capable of achieving submicron precision. Moreover, the combination of multi-material printing techniques will further enhance not only resolution but also allow for precise control of material distribution, enabling the creation of more complex gradient structures and functional designs. Multi-material integrated printing can create regions with varying mechanical properties, simulating the complexity of natural tissues, and further improving the functionality of bone scaffolds. Future biomanufacturing technologies are expected to overcome current resolution bottlenecks, offering more precise and efficient bone scaffold printing solutions.

Ensuring the safe clinical application of biodegradable scaffolds requires thorough evaluation of their degradation products. These products must undergo extensive biocompatibility and toxicity testing to ensure they do not trigger immune or toxic reactions. Biodegradable scaffolds typically degrade through processes such as hydrolysis or enzymatic breakdown, with their byproducts being excreted through metabolic systems like the liver and kidneys (Klabukov et al., 2023). However, in the case of highly porous scaffolds or large-scale implants, degradation products may accumulate locally, potentially creating an acidic environment or triggering inflammatory responses. To avoid these complications, scaffold material design must carefully balance degradation rates and the pathways for byproduct elimination, ensuring that the byproducts can be efficiently cleared by the metabolic system, preventing prolonged retention. The safety of degradation products can be assessed by regularly monitoring the concentration of metabolites in patient fluids (such as blood and urine) to track the degradation process and excretion efficiency. If degradation products accumulate or are excreted slowly, scaffold design may need to be adjusted, or more easily metabolized materials selected. Additionally, local accumulation of degradation products could trigger toxic reactions, making extensive animal testing and long-term human monitoring necessary during clinical trials to ensure these byproducts do not cause uncontrollable side effects.

Currently, bioprinting methods perform well in laboratory settings and for small-scale production, but when it comes to large-scale, complex scaffold printing, a major challenge is ensuring uniform cell distribution and effective nutrient diffusion (Shao et al., 2020). Complex scaffolds often have porous structures with high spatial resolution, which, while beneficial for cell proliferation and tissue growth, can also lead to uneven cell distribution or block nutrient and oxygen permeation due to closed pore structures. To address this issue, scaffold design needs to optimize pore structure and distribution. First, CAD can precisely control porosity and pore size, ensuring that the internal channels of the scaffold are large enough to facilitate cell migration and effective nutrient diffusion. Second, during the printing process, controlling the cell density and distribution in the bio-ink layer by layer can lead to more even initial cell distribution within the scaffold. Additionally, the microstructure of the scaffold should be designed with nutrient diffusion pathways in mind, avoiding complex or closed structures that could hinder nutrient transport. To further enhance cell infiltration and vascularization, the scaffold can be combined with bioactive coatings or embedded with microchannel structures. These measures help maintain uniform cell distribution during bioprinting, ensuring adequate nutrient and oxygen supply, and thereby promoting uniform bone tissue regeneration.

## 5 Conclusion

Bone tissue engineering holds significant potential in bone regeneration. This paper focuses on the fabrication of bone tissue engineering scaffolds, highlighting the material characteristics of scaffolds and the application of 3D bioprinting technology in this field. Through a comparison of different scaffold materials, we found that no single biomaterial possesses all the necessary properties for functional tissue reconstruction. The integration of 3D bioprinting technology has introduced new advancements in the field of bone tissue engineering. Although researchers have successfully utilized 3D bioprinting to construct certain bone tissue engineering scaffolds, clinical application remains in its early stages and faces significant challenges. Therefore, the development of bone tissue engineering scaffolds can be explored from the following three directions:

(1) The development of novel biodegradable bone tissue engineering scaffolds and continuous research into new scaffold materials are critical for advancing the field. As tissue engineering evolves, scaffold materials have gradually shifted from single to composite materials. Biodegradable scaffolds offer significant advantages by reducing the risk of secondary surgeries and infections, thereby promoting more efficient bone injury repair. Therefore, identifying and developing new biodegradable bone tissue engineering scaffolds is of paramount importance. In addition to the scaffold’s degradability, factors such as biocompatibility and the ability of cells to adhere to and proliferate on the scaffold surface must be considered. Researchers should continue to explore and develop new materials that enhance scaffold performance across various aspects, making them more suitable for clinical applications.
(2) Combining medical imaging technologies, such as CT and MRI, with 3D bioprinting can not only reduce preparation time before implantation but also enable the tracking of bone regeneration progress in patients. Imaging the anatomical structure of the target tissue through CT or MRI is the first essential step before 3D bioprinting, and the clarity and accuracy of these images directly influence the performance of the printed scaffold. Incorporating substances into the printing materials that can be monitored through CT or MRI would allow clinicians to track the degradation of the scaffold and observe the bone tissue regeneration process or cell proliferation. As the scaffold materials degrade, detecting signals from the incorporated substances could provide real-time monitoring of bone reconstruction and cell activity *in vivo*. Thus, integrating and advancing medical imaging technologies in 3D bioprinting plays a critical role in the development of bone tissue engineering scaffolds. Experts and scholars still need to conduct in-depth research.
(3) Exploring more efficient 3D printing methods is crucial to meet the diverse needs of different bone implant environments. Different patients often require varied treatment approaches, and the scaffolds they need must carry distinct biomaterials depending on the implantation environment and method. Factors such as the specific location of the implant, its biomechanical demands, and the required biological properties all influence the preparation of scaffolds. Therefore, future research should focus on developing more efficient and adaptable 3D printing techniques to address these complex and varying conditions.With the continuous improvement and refinement of 3D bioprinting technology and materials, 3D bioprinting is expected to bring profound changes to tissue engineering.

## Data Availability

The original contributions presented in the study are included in the article/supplementary material, further inquiries can be directed to the corresponding author.
